# Emerging Mycotoxins in Aquaculture: Current Insights on Toxicity, Biocontrol Strategies, and Occurrence in Aquafeed and Fish

**DOI:** 10.3390/toxins17070356

**Published:** 2025-07-17

**Authors:** Patrizio Lorusso, Giusy Rusco, Alessio Manfredi, Nicolaia Iaffaldano, Angela Di Pinto, Elisabetta Bonerba

**Affiliations:** 1 Department of Veterinary Medicine, University of Bari Aldo Moro, 70010 Valenzano, Italy; patrizio.lorusso@uniba.it (P.L.); angela.dipinto@uniba.it (A.D.P.); elisabetta.bonerba@uniba.it (E.B.); 2 Department of Agricultural, Environmental and Food Sciences, University of Molise, 86100 Campobasso, Italy; giusy.rusco@unimol.it (G.R.); nicolaia@unimol.it (N.I.)

**Keywords:** aquaculture, emerging mycotoxins, toxicity, occurrence, biocontrol strategies

## Abstract

Mycotoxins are secondary metabolites produced by various fungal species that can contaminate food and feed, posing significant risks to human and animal health. In aquaculture, the replacement of fishmeal with alternative protein sources has increased the risk of mycotoxin contamination, becoming a major challenge in fish feed production. Current data highlights that fish are exposed not only to common mycotoxins but also to emerging ones, raising concerns about human exposure through fish consumption. In this review, we draw attention to the toxicity data of key emerging mycotoxins from *Fusarium* (enniatins, ENNs; beauvericin, BEA) and *Alternaria* (alternariol monomethyl ether, AME; alternariol, AOH), their occurrence in aquafeeds and in commercially relevant fish species in Europe, and potential biocontrol approaches to prevent/mitigate contaminations. From the present review, it emerged that these mycotoxins exhibit in vitro cytotoxic properties. Their prevalence and concentrations vary widely both among aquafeeds, depending on the sample’s origin, and among fish species. Biocontrol approaches using microorganisms or natural compounds show promise as sustainable solutions to limit contamination. However, further research is essential to address data gaps and to allow for a proper risk assessment and, if necessary, the implementation of effective management measures.

## 1. Introduction

Mycotoxins are harmful secondary metabolites produced by multiple fungal species whose growth relies on favorable environmental conditions. Exposure to mycotoxins is a worldwide concern, and their occurrence is unavoidable and varies among geographical areas. They frequently affect cereal grains, in turn contaminating food and feed products made from them. In addition to the direct consumption of contaminated cereal-based foods, the potential carry-over of mycotoxins to animal byproducts, including eggs, meat products, and dairy products, also represents a food safety concern, as they can be taken up by humans as a consequence of an animal eating contaminated feed [1,2,3,4]. Feed contamination by mycotoxins is also a serious problem for livestock health, including fish [5,6].

Currently, in fact, mycotoxin contamination represents a significant challenge in aquaculture feed production, as these toxic fungal metabolites contribute to production losses by reducing body weight, growth performance, feed conversion efficiency, and immunity, while also causing organ damage and mortality in fish [7]. The increased risk of contamination is largely linked to the growing use of plant-based ingredients, such as corn, wheat, soybeans, and barley, as alternatives to traditional fishmeal, in efforts to cut costs and improve sustainability. Although more affordable and widely available, these ingredients are more susceptible to fungal contamination, increasing the risk of mycotoxicosis [7,8]. This risk is further exacerbated by inadequate post-harvest practices and poor storage conditions, especially in developing countries, where there is a lack of stringent regulations, monitoring, and surveillance systems [9,10]. As a result, aquafeeds frequently carry a range of mycotoxins posing threats not only to fish health and farm productivity but also to food safety due to potential carry-over into edible tissues [9].

Insects have also shown great potential as fishmeal substitutes in aquafeeds [11]. While insects themselves are not considered a direct risk to animal or human health, the primary concern for mycotoxin contamination stems from the substrates used to rear them [12,13].

The data on mycotoxin occurrence in fish feeds [14] and carry-over in various fish organs [15] shows that fish are at high risk of contamination, not only from mycotoxins for which maximum levels are set by the Regulation (EU) 2023/915 and subsequent amendments and additions, but also from emerging ones. These are a group of mycotoxins for which there are no available legislations or guidelines and no established maximum levels in crops and feeds in Europe and other parts of the world [6,16,17]; nonetheless, the evidence of their occurrence is a growing issue. *Fusarium* and *Alternaria* genera are known to be among the fungi able to colonize fish feed [18,19], as well as producers of emerging mycotoxins, such as *Fusarium* mycotoxins beauvericin (BEA) and enniatins (ENNA, ENNA1, ENNB, ENNB1), and *Alternaria* mycotoxins alternariol monomethyl ether (AME) and alternariol (AOH) [17]. Recent global studies have detected these mycotoxins in aquafeeds, with some also found in edible fish tissues, sometimes at high prevalence and concentrations, raising concerns about potential human exposure through fish consumption [6,15,20,21,22,23,24,25]. This concern is further heightened by a study carried out by Castell et al., which revealed the bioaccumulation of BEA, ENNA, ENNB, ENNA1, and ENNB1 in various human tissues, including brain, lung, kidney, fat, liver, and heart [26].

Although the European Food Safety Authority (EFSA) stated that the acute exposure to ENNs and BEA does not constitute a hazard to human health and does not define an acute reference dose or a tolerable daily intake for these secondary metabolites, their chronic exposure is still under study [27]. Estimating risk requires sufficient knowledge of the frequency with which mycotoxins occur; therefore, the data reported by Castell et al. improves the understanding of actual *Fusarium* emerging mycotoxin exposure levels, allowing for a more accurate update of the risk evaluation by the EFSA. On the other hand, to the best of our knowledge, no data is available regarding the bioaccumulation of *Alternaria* mycotoxins (AOH and AME) in human tissues, although recent in vitro studies show that both mycotoxins exert cytotoxic activities against tumor and normal cell lines (see Section 2.3).

The present review aims to summarize the current state of knowledge on the toxicity data of the main emerging mycotoxins of the *Fusarium* (ENNs and BEA) and *Alternaria* (AME and AOH) genera, including their occurrence in aquafeeds and in the most commercially significant fish species in Europe. Moreover, current biocontrol strategies to prevent/mitigate the contamination of fish feed commodities and the final products of mycotoxigenic fungi and emerging mycotoxins are highlighted. Our intent is to provide an updated overview of this research field that will help to identify the current data gaps and push the scientific community to close them in the coming years to allow for a proper risk assessment and, where necessary, propose specific and adequate management measures.

## 2. Toxicity

Several studies have investigated the toxicity of BEA, ENNs, AOH, and AME using both in vitro and in vivo models. However, most of the available scientific data come from in vitro studies, which provide valuable insights into the risk assessment associated with the presence of these mycotoxins in food and feed. Therefore, the following sections will present the latest research on the toxicity of these mycotoxins in various cell lines, along with the underlying mechanisms responsible for their effects.

### 2.1. Beauvericin

The cytotoxic effects of BEA have been described in different in vitro studies involving several cell lines. Prosperini et al. showed that BEA induced a significant decrease in human colon adenocarcinoma (Caco-2) cell viability in a dose- and time-dependent manner. Caco-2 cells were treated with BEA at concentrations ranging from 3.125 to 25 μM and monitored after 24, 48, and 72 h. The IC_50_ values obtained after BEA exposure ranged from 3.2 ± 1.1 to 20.6 ± 6.9 μM, as determined by the MTT assay, and from 1.9 ± 0.7 to 8.8 ± 0.9 μM, as determined by the NR assay [28]. Similarly, BEA has been shown to induce cytotoxicity in Chinese Hamster ovary (CHO-K1) cells after 24, 48, and 72 h of exposure, with IC_50_ values of 10.7 ± 3.7, 2.5 ± 3.3, and 2.2 ± 3.3 μM [29]. On the same cell line, Ferrer et al. reported IC_50_ values of 17.22 ± 1.20 μM and 12.08 ± 1.10 μM after 24 h of treatment with BEA concentrations ranging from 1 to 300 μM, as determined by the NR assay and the MTT assay, respectively [30].

BEA treatment demonstrated a time-dependent reduction in the survival of human NSCLC A549 cells at concentrations of 10 and 30 μM. At 10 μM, significant cell death was observed after 3 h of exposure, with cell survival declining to less than 20% after 24 h. At 30 μM, a decrease in cell survival was evident within 1 h and dropped sharply after 3 h of incubation. The IC_50_ value for BEA after 24 h of treatment was calculated to be 4.5 ± 0.35 μM [31]. Wätjen et al. performed cytotoxicity experiments on four different cell lines: rat hepatoma (H4IIE), human hepatoma (HepG2), human colon carcinoma (Hct116), and rat glioma (C6) cells. After 24 h of BEA treatment, the IC_50_ values were approximately 1.9 μM for H4IIE, 1.0 μM for C6, 2.4 μM for Hct116, and 3.6 μM for HepG2 [32]. Recently, two additional studies were carried out on HepG2 cells. In the first, this cell line was treated with BEA concentrations from 0 to 25 μM for 24, 48, and 72 h, obtaining IC_50_ values of 12.5 ± 0.04 μM, 7.01 ± 0.05 μM, and 5.5 ± 0.07 μM [33]. In the second, on the other hand, HepG2 cells were treated with BEA at concentrations between 0.1 μM and 100 μM, observing an important decrease in viability already at concentrations of 0.1 μM and 1 μM [34]. Fraeyman et al. examined the toxicity of BEA on proliferating and differentiated porcine intestinal epithelial cells (IPEC-J2). They demonstrated that a 5 μM concentration of BEA reduced the viability of proliferating cells to 82%, while a 10 μM concentration decreased the viability of differentiated cells to 47% [35]. Manyes et al. investigated the effects of BEA on the immune system by exposing human T lymphoma cell lines (Jurkat T-cells), derived from acute T-cell leukemia, to concentrations ranging from 1 to 15 μM for 24, 48, and 72 h. They reported IC_50_ values of 7.5 ± 0.4 μM at 24 h, 5.0 ± 0.3 μM at 48 h, and 3.0 ± 0.6 μM at 72 h [36]. Similarly, Ficheux et al. evaluated the toxicity of BEA on other immune cells, including immature dendritic cells, mature dendritic cells, and macrophages. Exposure to 6.4 μM and 32 μM of BEA resulted in cell survival rates of 9%, 24%, and 8%, respectively. The IC_50_ values were 1.0 μM for immature dendritic cells, 2.9 μM for mature dendritic cells, and 2.5 μM for macrophages [37]. Finally, in a study conducted by Schoevers et al., it was shown that BEA is also capable of exerting toxic effects on embryos, oocytes, and cumulus cells when exposed to concentrations above 0.5 μM of this mycotoxin [38]. Therefore, BEA demonstrates strong cytotoxicity across diverse cell types, significantly reducing viability at low micromolar concentrations and showing pronounced effects on immune, hepatic, and reproductive cells. The mechanisms underlying the cytotoxic effects of this mycotoxin are currently under investigation; however, nowadays, several studies have shown how BEA is able to promote cell cycle arrest, apoptosis, and the generation of reactive oxygen species (ROS), leading to lipid peroxidation and a decrease in antioxidant enzymes [28,29,30,31,32,36,39,40,41,42,43].

Moreover, the EFSA assessed the genotoxicity of BEA, considering studies suggesting DNA damage as a secondary consequence of oxidative stress and apoptosis induced by this mycotoxin. However, after a thorough review of the available data, the EFSA concluded that there is no evidence of direct genotoxic effects associated with BEA. Instead, DNA damage is primarily linked to indirect mechanisms, notably mitochondrial dysfunction [44].

### 2.2. Enniatins

As for BEA, there are several studies that describe the in vitro cytotoxicity of ENNs. Prosperini et al. investigated the cytotoxic effects of ENNA, ENNA1, ENNB, and ENNB1 at concentrations ranging from 0.45 to 15 μM on Caco-2 cells after 24, 48, and 72 h of exposure, using MTT and NR assays. For all the tested mycotoxins, a dose- and time-dependent decrease in cell viability was observed. ENNA1 and ENNA exhibited the highest cytotoxicity, followed by ENNB1 and ENNB, which showed the lowest cytotoxic effects [45]. Fernández-Blanco et al. treated the same cell line with ENNB concentrations ranging from 0.312 to 10 μM, obtaining similar results. Specifically, ENNB did not produce an IC_50_ value within the tested concentration range after 24 and 48 h of treatment. However, an IC_50_ value of 3.9 μM was observed after 72 h [46]. Meca et al. (2011) [47] investigated the hepatotoxicity of these mycotoxins by exposing HepG2 cells to concentrations of ENNA, ENNA1, ENNB, and ENNB1 ranging from 0.6 to 30 μM for 24 and 48 h. Similar to their effects on Caco-2 cells, ENNA1 was the most cytotoxic enniatin tested, with IC50 values of 11.6 ± 5.7 μM after 24 h and 2.6 ± 0.6 μM after 48 h of exposure. ENNB1 followed with IC_50_ values of 23.4 ± 7.6 μM and 8.5 ± 3.7 μM after 24 and 48 h, respectively. ENNA exhibited IC_50_ values of 26.2 ± 7.6 μM and 11.4 ± 4.6 μM over the same time periods. No IC_50_ value could be determined for ENNB within the tested concentration range. In a related study, ENNA1 was shown to significantly reduce HepG2 cell viability at very low concentrations (0.1 and 1 μM), further supporting its potential hepatotoxic effects [34]. Fraeyman et al. experimented with the toxicity of ENNs on IPEC-J2 intestinal cells by exposing them to ENNA, ENNA1, ENNB, and ENNB1 at concentrations ranging from 0 to 100 μM. They observed that a 10 μM concentration of ENNA reduced the viability of proliferating cells to 30% and differentiated cells to 36%. At the same concentration, ENNA1 and ENNB1 reduced the viability of proliferating and differentiated cells to 86% and 93%, respectively. In contrast, ENNB at 25 μM decreased the viability of proliferating and differentiated cells to 92% and 89%. Based on these findings, the authors concluded that the relative in vitro cytotoxicity toward both proliferating and differentiated IPEC-J2 cells could be ranked as ENNA >> ENNA1 > ENNB1 >> ENNB [35]. Krug et al. studied the possible neurotoxic effects of ENNB and ENNB1 using different blood–brain barrier cells: porcine primary cerebral capillary endothelial cells (PBCECs), human cerebral microvascular capillary cells (HBMECs), and CCF-STTG1, an astrocytoma cell line. The cells were exposed to concentrations of these mycotoxins ranging from 0.1 μM to 10 μM for 48 h. In summary, no significant changes in HBMEC cell viability were observed for either mycotoxin within the concentration range of 0.1 μM to 10 μM. In the PBCEC cells, ENNB caused notable cytotoxicity at concentrations above 5 μM, reducing cell viability to as low as 70%. ENNB1 demonstrated slightly greater cytotoxicity, significantly reducing cell viability at concentrations of 2.5 μM and higher, with a relative viability of 64% at 10 μM. Finally, in the CCF-STTG1 cells, ENNB caused significant cytotoxic effects at 5 μM and 10 μM, whereas ENNB1 produced mild but significant effects at 0.1 μM and 1 μM, with strong cytotoxicity observed at concentrations starting from 2.5 μM. The IC_50_ values were determined to be 8.9 μM for ENNB and 4.4 μM for ENNB1 [48]. In two studies, the effect of ENNs on immune system cells was evaluated. In the first, immature dendritic cells, mature dendritic cells, and macrophages were exposed to concentrations of ENNB ranging from 0.1 to 10 μM, and the IC_50_ values calculated were 1.6 μM, 2.6 μM, and 2.5 μM, respectively [37]. In the second, Jurkat T-cells were exposed to ENNB at concentrations ranging from 1 to 15 μM for 24, 48, and 72 h. The treatment resulted in a reduction in cell viability, with decreases of 21% observed after 24 h, 23% after 48 h, and 29% after 72 h [36].

The studies reviewed indicate that ENNs exhibit varying levels of cytotoxicity across different cell types, with effects generally dependent on dose and exposure time. Their cytotoxic mechanisms include reduced cell viability, cell cycle arrest, apoptosis induction, and elevated ROS production [34,35,36,45,48,49,50].

Moreover, similar to BEA, the genotoxic potential of ENNB has also been studied. In this context, the EFSA, in a Scientific Report published in 2018, concluded that ENNB may exert genotoxic effects, particularly targeting bone marrow and liver cells [27].

### 2.3. Alternaria Toxins

The first risk assessment on *Alternaria* toxins was carried out in October 2011, when the EFSA published a Scientific Opinion on the risks to animal and public health associated with their presence in food and feed [51]. Since then, several studies have explored the toxicity of AOH and AME in different cancerous and normal cell lines.

Ishikawa cells, a well-established model derived from endometrial adenocarcinoma, were exposed to AOH at concentrations of 1, 2.5, and 5 μM for 48 h. It was observed that cell viability remained unchanged at 1 and 2.5 μM, while the highest concentration tested caused a reduction in cell growth by up to 63.5% [52]. Den Hollander et al. examined the cytotoxicity of AOH and AME on HepG2 and Caco-2 cells, testing a concentration range from 0.1 to 120 μg/mL. After 24 h of exposure, HepG2 cells exhibited EC_50_ values of 11.68 ± 4.05 μg/mL for AOH and 5.07 ± 0.52 μg/mL for AME. In contrast, 48 h of exposure to AOH and AME in Caco-2 cells resulted in EC_50_ values of 18.71 μg/mL and 15.38 ± 8.62 μg/mL, respectively. These findings indicate that AME exerts stronger cytotoxic effects than AOH on both cell lines [53]. Mahmoud et al. conducted a similar experiment on the same cell line, exposing HepG2 cells to varying concentrations of AOH and AME, ranging from 0 to 100 μg/mL [54]. Consistent with the findings of Den Hollander et al., AME demonstrated greater cytotoxicity, with an EC_50_ of 9.80 μg/mL, compared to an EC_50_ of 28 μg/mL for AOH. Further investigation by the same authors into the effects of AME on A549 and PC-3 cell lines revealed an EC_50_ of 0.73 μg/mL for A549 cells and 0.17 μg/mL for PC-3 cells, underscoring the cytotoxic nature of this mycotoxin [54]. In a separate study, PC-3 cells were exposed to AOH at concentrations ranging from 0.001 to 100 μM for 24 and 48 h. Similar to AME, AOH also exhibited cytotoxic effects on this cell line, with a complete cell viability reduction observed at concentrations of 30 μM and higher at both time intervals [55]. Additionally, Kozieł et al. investigated the toxicity of AOH on human ovarian carcinoma SKOV-3 cells by exposing them to the same concentration range (0.001 to 100 μM) for 24 h. Their findings revealed an IC_50_ value of 52.68 μM, which aligns with values reported in other studies involving different cancer cell lines [56].

Similar experimental models have been employed to investigate the cytotoxic effects of AOH and AME on normal cell lines. Lin et al. (2023) exposed human gastric epithelial cells (GES-1) to six different concentrations of AOH and AME (0.25–60 μM) for 48 h, determining IC_50_ values of 14.84 μM and 10.21 μM, respectively [57]. In a separate investigation, treatment with 4 ppm of AOH reduced GES-1 cell viability to 82%, further underscoring the susceptibility of this cell line to these mycotoxins [58]. Finally, Urbanek et al. examined the cytotoxicity of AOH on human prostate epithelial cells (PNT1A), revealing that concentrations of 30 μM or higher completely reduced cell viability after 24 and 48 h of exposure [55].

Therefore, studies in the recent scientific literature show how both mycotoxins exert cytotoxic activities against both tumor and normal cell lines.

The cytotoxic mechanisms of these mycotoxins are still under investigation. However, the current research has demonstrated that they induce DNA damage by causing single- and double-stranded breaks, along with oxidative damage resulting from reactive oxygen species (ROS) generated through their metabolic byproducts, such as catechols and quinones [59]. Additionally, AOH and AME have been shown to inhibit topoisomerase activity, stabilizing the enzyme–DNA complex and leading to DNA breaks [54]. This damage activates the DNA damage response pathway, involving key proteins, such as p53, PCNA, and p21, which contribute to cell cycle arrest, autophagy, and senescence [59].

## 3. Occurrence of BEA, ENNs, AOH, and AME in Aquafeed

Global fish consumption has been on the rise due to increasing demand, world market availability, and consumer trends related to concerns for healthy eating. In 2022, global aquaculture production reached 130.9 million tonnes, with a total value of USD 312.8 billion. Looking ahead, aquatic animal production is projected to grow 10% through 2032, fueled by the continued expansion of aquaculture and the recovery of capture fisheries. By then, total production is expected to reach 205 million tonnes, with aquaculture contributing 111 million tonnes [60].

This expansion is reflected in the demand for fish feed; however, the limited availability of marine raw materials, such as fishmeal and fish oil, has forced the European aquafeed industry to explore innovative and sustainable alternative sources of protein and lipid for feed production, like plant-based ingredients, introducing new types of contaminants into the aquaculture sector [23]. Consequently, growing concerns about potential risks to both human and animal health have heightened interest in evaluating contaminants in aquaculture feed formulations. In this context, mycotoxins are recognized as potential contaminants in plant-based fish feed, as they naturally occur in these raw materials [25].

Until now, emerging mycotoxins in fish feed have been reported in only a few studies, as listed in Table 1. In a study carried out by Søderstrøm et al., Norwegian samples, including 77 plant-based meals, 51 plant-based oils intended for fish feed production, and 200 fish feed samples, were analyzed for both well-known and emerging mycotoxins. Among all matrices analyzed, ENNB was the most frequently detected mycotoxin, showing the highest prevalence rates: 80% in fish feed, 15% in plant proteins, and 88% in plant oils. It was also found at the highest concentrations in fish feed and plant oils, reaching levels of 250 and 450 μg/kg, respectively. In contrast, BEA was the most concentrated mycotoxin detected in plant proteins, with a value reaching 2400 μg/kg. Furthermore, separate analysis of the feed ingredients allowed the authors to identify rapeseed oil, as well as wheat and corn gluten, as major sources of ENNB. Corn gluten was also identified as the primary source of BEA [61].

Consistent with the findings of Søderstrøm et al., Mwihia et al. reported ENNB as the most prevalent mycotoxin among 78 analyzed fish feed samples, followed by BEA, ENNB1, AOH, ENNA1, ENNA, and AME. Although ENNB was the most frequently detected, BEA was present at the highest concentrations, reaching a maximum level of 841.8 μg/kg in one sample [24]. Similarly, in a study by Tolosa et al. that analyzed 20 fish feed samples, ENNB, alongside ENNB1, ENNA, and ENNA1, showed the highest prevalence (100%), while BEA was detected in 95% of the samples. Moreover, ENNB1 exhibited the highest concentration levels among the detected mycotoxins, reaching up to 10 μg/kg [62]. The high prevalence rates reported in their study were attributed to the exceptional sensitivity of the analytical methods used. As shown in Table 1, the technique employed by the authors allowed for lower limits of detection (LOD) and quantification (LOQ) compared to those used in previous studies. This enabled the detection of even very low concentrations of the targeted mycotoxins, providing a more accurate estimation of the true level of contamination in the samples. Finally, Albero et al. analyzed nine rainbow trout feed samples and detected ENNB, ENNB1, and BEA in all of them. Consistent with previous findings, BEA was present at the highest concentration levels, reaching a value of 30 ± 2 μg/kg, followed by ENNB and ENNB1, which were detected at maximum concentrations of 21 ± 1 μg/kg and 7.9 ± 1.2 μg/kg, respectively [25].

Overall, these studies collectively highlight the widespread occurrence of emerging mycotoxins in fish feed and its ingredients, with ENNB consistently emerging as the most frequently detected compound across various matrices. BEA, while less prevalent, often appears at the highest concentrations, suggesting it plays a notable role in overall contamination patterns.

This scenario reflects a broader global trend; according to Gruber-Dorninger et al., 98.7% of fish feed samples worldwide are contaminated with at least one mycotoxin, 75.7% with five or more, and 39.4% with ten or more, underscoring the high complexity of co-occurrence and potential additive or synergistic effects among multiple toxins [63].

A significant limitation associated with these mycotoxins is the lack of a comprehensive risk analysis necessary to establish maximum levels in aquafeeds. To date, European regulatory limits have been established only for aflatoxin B1 (AFB1), with maximum levels set at 0.02 mg/kg in raw materials and 0.01 mg/kg in complementary and complete feeds [64]. Although it would not be appropriate to directly compare emerging mycotoxins to these thresholds, since the AFB1 maximum levels are based on a dedicated risk analysis, it is noteworthy that emerging mycotoxins are often detected at concentrations substantially higher than the established maximum levels for AFB1.

The findings, moreover, underscore the critical role of highly sensitive analytical techniques in detecting even low concentrations of emerging mycotoxins, thereby enabling a more accurate assessment of contamination and supporting the development of future regulatory frameworks for aquafeed safety.

With Regulation EU No. 2017/839, the implementation of meals from insect species such as the black soldier fly (*Hermetia illucens*), the common housefly (Musca domestica), the yellow mealworm, the lesser mealworm (*Alphitobius diaperinus*), and crickets (*Acheta domesticus*, *Gryllodes sigillatus*, and *Gryllus assimilis*) in aqua feed formulations was officially authorized [65].

Although insects are not highlighted in the literature when the goods or animals most affected by the effects of mycotoxins are mentioned, recent studies confirmed that the substrates used for raising and breeding insects can also contain contaminants and secondary toxic metabolites, such as common and emerging mycotoxins, which, in turn, can accumulate in farmed insects [12,13,66]. To date, the reported accumulation and metabolism patterns of contaminants strongly depend on the insect species and the developmental stage of the insects, emphasizing the importance of assessing potential safety hazards on a case-by-case basis. In light of these considerations, similar to plant-based protein sources, the consequences of this problem on fish and human consumption deserve attention, and their clarification by primary studies will be relevant for its mitigation. In the meantime, in order to limit the potential mycotoxin contamination of fish feed resulting from the use of insect-based meals, it is recommended to regularly monitor contaminants in rearing substrates and to use insects that have followed a fasting period of at least 24 h prior to harvest [13,66].

## 4. Occurrence of BEA and ENNs in Fish

In fish, the presence of mycotoxins in feed triggers various disorders by disrupting nutrient absorption, damaging cells and organs, and causing both functional and morphological changes. In severe cases, these effects can lead to mortality. As a result, mycotoxin contamination in feed contributes to substantial aquaculture losses both directly through increased mortality and indirectly by reducing production and promoting secondary diseases [67,68,69]. For example, in a sub-chronic, three-month feeding trial with pre-smolt Atlantic salmon, dietary BEA led to a significant decrease in protein digestibility and feed-conversion efficiency, resulting in impaired growth, while ENNB produced stunted growth and anemia; both toxins promoted oxidative stress, evidenced by depletion of hepatic vitamin E and an increased incidence of spinal deformities [70]. In a separate acute exposure study, non-lethal doses of ENNB activated inflammation-related pathways in the intestine, suggesting epithelial damage and immune activation, whereas BEA caused hepatic hematological disruptions associated with heme metabolism and oxidative stress [61].

Studies offer conflicting views on the carry-over of emerging mycotoxins in fish [62,71]. Nácher-Mestre et al. investigated the prevalence and levels of BEA, ENNA, ENNA1, ENNB, and ENNB1 in fish feeds, as well as in whole fish and fillets of sea bream and Atlantic salmon fed with those feeds. Although BEA, ENNA1, ENNB, and ENNB1 were detected in the feeds at concentrations of up to 80.4, 3.8, 32.8, and 10.9 μg/kg, respectively, none of the whole fish or fillet samples contained the parent compounds of the studied mycotoxins. However, the authors did not rule out the possibility that these molecules could have been metabolized and/or accumulated in specific organs at concentrations below the detection limits of the analytical methods used. This observation further highlights the importance of sensitive analytical techniques, as discussed in the previous section, in providing a more accurate estimation of the true level of contamination in the analyzed samples [23].

On the contrary, studies performed by Tolosa et al. confirmed the presence of these emerging mycotoxins in these organisms [21,62,72]. Specifically, as illustrated in Table 2, the studies investigated the occurrence of these mycotoxins in samples of sea bass (*Dicentrarchus labrax*), sea bream (*Sparus aurata*), Atlantic salmon (*Salmo Salar*), and Rainbow Trout (*Oncorhynchus mykiss*), which are among the most commercially significant in Europe [60]. In sea bass and sea bream, ENNA and BEA were not detected. However, ENNA1, ENNB, and ENNB1 were found at varying concentrations and prevalence levels. In sea bass, ENNA1 levels ranged from 1.7 to 6.9 μg/kg, with a prevalence of 50%; ENNB was detected with values varying significantly (up to 44.6 μg/kg in one study and up to 12.8 μg/kg in the other) and a high prevalence of 90%, while ENNB1 ranged between 1.4 and 31.5 μg/kg, with a prevalence of 70%. In sea bream, although the concentration values were generally lower than in sea bass, ENNA1 was still present (2.1–7.5 μg/kg, 30% prevalence), along with ENNB (1.3–21.6 μg/kg, 40% prevalence) and ENNB1 (7.1–19 μg/kg, 30% prevalence).

In Atlantic salmon, ENNA and BEA were not detected, whereas ENNs (ENNA1, ENNB, and ENNB1) were found at higher concentrations despite a relatively low prevalence of 20%. In rainbow trout, only ENNB and ENNB1 were detected, with single values of 3.6 and 2.9 μg/kg, respectively, and a low prevalence of 10% [21,62].

It is noteworthy that BEA was not detected in any of the analyzed species, suggesting that, based on the results obtained from these studies, BEA may not represent a significant contamination concern in the fish examined. However, further investigations are needed to confirm this finding.

The significant variability in emerging mycotoxin concentrations and prevalence found in fish could be attributed to the different degrees of species-specific susceptibility, as already observed for some regulated mycotoxins [8,14,73]. In order to enable a more reliable risk assessment, future studies should focus on in vivo dose–response exposure tests of the same mycotoxin on different fish species under the same experimental conditions to gain more detailed information required to (1) derive reliable recommendations to establish maximum levels of these mycotoxins in fish feeds; (2) tailor mitigation strategies; and (3) optimize the selection of fish species in aquaculture operations.

Moreover, rigorous ongoing monitoring and the adoption of standardized analytical methods are needed. This is critical because even minor fluctuations in mycotoxin levels can have profound effects on fish health, potentially compromising their immune systems, growth rates, and overall well-being [74]. Such adverse impacts not only affect the productivity of aquaculture operations but also pose serious health risks to consumers through the potential transfer of these toxins via the food chain. Therefore, implementing uniform testing protocols and regular surveillance is essential to safeguard both animal welfare and public health, ultimately ensuring the quality and safety of aquaculture products.

## 5. Prevention and Detoxification Strategies of Emerging Mycotoxins in Aquafeeds with Special Focus on the Biocontrol Strategies

The main fungal genera from which the mycotoxins commonly detected in aquafeeds are produced are *Aspergillus* spp., *Fusarium* spp., *Penicillium* spp., and *Alternaria* spp. [18,19,75,76,77,78,79]. Climatic, genetic, and geographic factors can affect the occurrence and concentration of mycotoxins in different feed raw materials and finished feeds [80,81,82,83]. At the same time, inadequate conditions of cultivation, harvesting, transportation, receiving, and storage and poor processing practices are among the most critical factors that contribute to the risk of mold production and, subsequently, of mycotoxins in feeds [78,84,85]. Moreover, climate change also plays a crucial role in driving the growth and development of fungal species and the emergence of new mycotoxins in commodities [86,87], highlighting the importance of adopting a “One Health” approach in accordance with that established by sustainable agriculture programs. In light of these considerations, it becomes clear that controlling mycotoxins in feeds is a big task, and the adoption of a single approach for this contamination is not enough. In fact, although preventive actions are the best way to mitigate mycotoxins in feeds, very often, the risk of fungal contamination is unavoidable; therefore, their control can be addressed using various conventional and innovative detoxification methods [86,88,89,90,91,92]. In the planning of any prospective intervention strategy for the management of mycotoxin risks in the feed supply chain, an integrated system based on the synchronized use of prevention, control, and detoxification tools is necessary through the adoption of Good Agricultural Practices (GAPs), Good Manufacturing Practices (GMPs), Good Hygienic Practices (GHPs), quality control, Hazard Analysis and Critical Control Point (HACCP), and strategies to counteract mycotoxins directly in feeds [86,93].

Numerous reviews have reported several strategies developed to date to help prevent the growth of mycotoxigenic fungi as well as to decontaminate and/or detoxify various mycotoxin-contaminated feed types and ingredients [86,88,90,91,92,94,95,96]. However, there is very limited and generalized information in the literature about effective approaches to manage fungi and mycotoxin contamination risks in aquafeed [97,98], especially when it comes to emerging mycotoxins, since the data on their occurrence, concentration levels, and toxicity in animals, including fish and humans, is still limited [6,14,62]. In fact, for risk characterization and assessment, accessible scientific knowledge, expert judgment, and the weight of evidence are essential, and, where necessary, specific risk management measures will be proposed, such as a proposal of laws and the establishment of maximum levels, techniques, and practices to reduce risks. Therefore, future studies will be indispensable in reducing data gaps and developing and implementing any suitable mitigation strategies. In the meantime, it may be useful to implement preventive measures to evade contamination by the main mycotoxigenic fungi found in aquaculture feed raw materials and finished feeds, as well as potential synthesis of common and emerging mycotoxins, as a curative method [99]. In this regard, the study of biological control methods with enhanced antifungal effectiveness to prevent contamination of agricultural commodities, feed, and food has become the new trend, driven by growing preference of consumers for the use of natural agents to the detriment of chemical ones, due to their positive impact on the environment, food safety, and food security [100,101,102]. As a potentially effective biocontrol strategy for the growth and sporulation of three fungal isolates (*Penicillium verrucosum*, *Aspergillus flavus*, and *Fusarium solani*), representative of those in fish feed, the inhibitory effect of volatile organic compounds (VOCs) emitted from a novel *Bacillus cereus* BC344–2 strain was recently tested [79]. The in vitro biocontrol co-incubation assay showed that BC344–2 VOCs had a significant inhibitory effect on fungal growth in terms of a decreased growth rate and a significant alteration of their sporulating capacity. *P. verrucosum* showed the highest sensitivity with a 42.4% inhibition ratio, followed by *F. solani* (17.5%) and *A. flavus* (11.5%). Additionally, BC344–2 VOCs suppressed the synthesis of OTA and AF mycotoxins by *P. verrucosum* and *A. flavus*, respectively.

Another promising biocontrol strategy to overcome fungal contamination in fish feed is exposure to smoldered fumes of specific plants. “Smoldering” refers to a slow, flameless, low-temperature form of combustion that occurs when oxygen reacts directly with condensed-phase biological materials in the presence of heat. This incomplete combustion process can release numerous volatile compounds with significant bioactivities [103]. In particular, Alasmari and Sakran proposed the biocontrol of aflatoxigenic *A. flavus* in fish feed by exposure to smoldering fumes of Cinnamon bark, Athl, and Lilac leaves. All treatments were successful in inhibiting *A. flavus* growth both in agar media and in contaminated fish feed. The Cinnamon treatment was the most effective and could entirely inhibit fungal growth at a proportion of 20 g/m^3^ both in Petri plates and in experimentally infested feed [104].

The use of isothiocyanates (ITCs), which are natural reactive compounds from plant belonging to the family Brassicaceae, has also been explored as a preventive strategy for the inhibition of mycotoxigenic fungi (*Aspergillus parasiticus* and *Fusarium poae*) in wheat flour [105] and as a mitigation method for reducing the emerging mycotoxin beauvercin (BEA) both in buffer solutions and in cereal grains and their flours [106,107]. Specifically, allyl isothiocyanate (AITC), a volatile antimicrobial derived from oriental and black mustard, was investigated to evaluate its ability to inhibit the mycotoxin production of *Aspergillus parasiticus* (AF producer) and *Fusarium poae* (BEA and ENNs producer) in wheat flour. Samples of flour contaminated with fungi were treated with variable concentrations (0.1, 1, and 10 µL/L) of AITC loaded on filter paper and inserted into hermetically closed jars. The AITC volatilized at room temperature was able to inhibit the production of all mycotoxins (AFs, BEA, and ENNs) in a dose-dependent manner, showing a total inhibition of mycotoxin synthesis for 30 days when applied at the highest concentration (10 µL/L).

The ability of the AITC reactant was also tested for reducing BEA at a concentration of 25 mg/kg in two experimental settings: a solution model (PBS at pH 4 and 7) and wheat flour [106]. In PBS, at both pH values, BEA disappeared rapidly in a time-dependent manner, averaging a reduction of 77% after 4 h, 85% at 8 h, 97.5% after 24 h, and 100% after 48 h. In wheat flour treated with gaseous AITC (50, 100, and 500 µL/L) in a hermetically closed glass jar, the BEA reduction levels ranged from 10% to 65% in a dose-dependent manner. In both experiments, the reduction in BEA was caused by the formation of two new reaction products, identified by LC-MS-LIT, corresponding to BEA conjugates containing one or two molecules of AITC. These conjugates were found to be nontoxic to Caco-2 cells (unpublished data cited in the review by Luz et al. [108]).

Luciano et al. tested the reaction of BEA (25 mg/kg) with two other types of ITCs, benzyl isothiocyanate (BITC) and phenyl isothiocyanate (PITC), in PBS (pH 4, 7, and 10) and in kernels and flours of different cereals (barley, wheat, rice, and corn). In the PBS buffer, the reduction in BEA ranged from 9% to 94% in a time-dependent fashion. An acid pH favored the reaction between the mycotoxin and the ITCs, leading to higher BEA reduction (94% and 81%, respectively, in PITC and BITC after 48 h) [107]. Comparing these results with those obtained by Meca et al., the BEA reduction with AITC was much faster, reaching 80% reduction after 4 h and a complete disappearance after 48 h at both pH 4 and 7 [109]. It is plausible that the short aliphatic chain of AITC facilitates the reaction of this compound with BEA in comparison with the more voluminous aromatic side chains of BITC and PITC. Treatments with the essential oils of PITC and BITC at different concentrations (50, 100, and 500 µL/L) were used to fumigate kernels and flours, which caused a pronounced reduction in BEA (ranging from 9% to 97%) in a dose-dependent fashion. Of all the grains tested, BEA was mostly affected by the action of the ITCs in the corn sample, followed by the wheat, rice, and barley samples. Overall, PITC demonstrated a greater reduction in BEA compared to BITC. For both gases, the reaction rates with BEA on the kernels were higher than those detected on the flours, probably because of their different physical structure. In fact, unlike the flours, the surface of the kernels could favor the direct contact of the gas with the mycotoxin, while the flours may represent a barrier to the penetration of the gas. In this regard, the use of forced air to push the ITCs through the flour could elicit higher rates of the ITC-BEA reaction. The degradation range of the mycotoxin BEA in wheat flour by AITC observed by Meca et al. is comparable with the reduction levels found for PITC and BITC after 48 h at 100 µL/L and 500 µL/L. Instead, at the lowest ITC reactant concentration (50 µL/L), AITC showed faster BEA reduction (46,6%) than PITC (17%) and BITC (8%) [109].

BEA has also been the target mycotoxin for testing some methods of biological degradation, using probiotic bacteria (only in vitro) [109], yeasts, and yeast enzymes in model solutions and in a food/feed system [110,111].

Meca et al. [109] evaluated, for the first time, the in vitro BEA reduction ability of 13 probiotic bacterial strains used as detoxification agents. In the growth mediums, the residual concentration of BEA, the toxin concentration adsorbed by bacteria on the cell wall, and also the BEA amount internalized in the cells, were analyzed by LC-MS/MS. All the bacteria evaluated showed a significant BEA reduction during the fermentation process, with a mean diminution variable ranging from 66 to 83%. The bacterium that recorded the highest reduction in the compound studied was the strain of *Lb. rhamnosus*. The results showed that this reduction was partly due to bacterial internalization and partly to adsorption of the mycotoxin by the cell wall. Mass spectrometry analysis demonstrated that BEA can interact with some component of the bacterial cell wall, such as phospholipids, potentially leading to a loss of its toxicity [109].

In a successive study, the interaction between BEA and nine yeast strains of *Saccharomyces cerevisiae* was tested in Potato Dextrose Broth (PDB) medium and in corn flour by the same authors [110]. The results showed that the bioactive compound BEA can be degraded in fermented PDB medium by all the tested strains of *S. cerevisiae* in 48 h, reducing the BEA concentration by an average of 86%. These results were validated in a real food/feed system composed of corn flour contaminated with 25 mg/kg of BEA. Here, all the strains tested confirmed a degradation activity toward BEA with a mean reduction of 71%. The highest reduction in BEA was 90.0 ± 3.1% by strain of *S. cerevisiae* A42.

Excellent results were also achieved when BEA degradation, in PBS and corn flour, was performed using intracellular raw enzymes extracted by four strains of *S. cerevisiae* [111]. In PBS, BEA reduction ranged from 83 to 99.3% in a time-dependent manner, reaching the highest degradation percentage after 24 h with enzymes of the A34 strain. The effectiveness of the enzymatic complexes tested was also confirmed in corn flour. Similar to that in PBS, the reduction rate increased with a prolonged incubation time from 68 to 91%. The highest BEA reduction was achieved by intracellular raw enzymes from the yeast strain LO9 after 24 h. After the BEA degradation by these microbial intracellular proteases, a new product was detected in both the model solution and the food system. The authors recommended studying the new product’s toxicity by employing cell models, with the aim of its future use in animal feed. In both of these last two studies, Meca et al. [110,111] revealed the potential mechanisms of BEA degradation by applying LC-MS-LIT, with a high precision level, to analyze the structure of newly formed compounds generated by BEA degradation due to yeasts and yeast enzymes of the *S. cerevisiae* A34 strain. In both cases, the chromatograms revealed the presence of new peaks and mass fragments corresponding to products with structures different from the original BEA, confirming its reduction and its structural modification during microbial biodegradation.

A summary of all the above-mentioned strategies is reported in Table 3. The outcomes indicate the potential utilization of bacterial isolates, volatile bioactive compounds from smoldering procedures, ITCs or ITC-containing products, probiotic bacteria, and yeast as promising natural agents in the preservation of fish feed commodities and final products against fungal attacks and accumulations, as well as the reduction or detoxification of mycotoxins, including emerging ones. With regard to potential applicability, volatile isothiocyanates act in the gaseous phase, making them technically compatible with sealed storage systems or packaging environments, while probiotic bacteria and yeast strains are already widely used in the feed industry for other purposes. Therefore, this existing regulatory acceptance and their compatibility with fermentation processes could facilitate their integration as functional additives for mycotoxin mitigation in feed. However, future investigations should assess the safety of the reaction products generated by the interaction between mycotoxins and the applied biocontrol or biodegradation agents through comprehensive in vitro and in vivo toxicity studies. Additionally, the effectiveness of these natural agents should be explored against other emerging mycotoxins beyond BEA, and their cost-effectiveness under real production conditions should be evaluated.

The European Union has already authorized the use of some natural substances as technological feed additives that are able to reduce mycotoxin contamination of feed by mechanisms that limit their absorption and bioaccessibility. Among these, *Eubacterium* strain BBSH 797 (microorganism strain DSM 11798 of the *Coriobacteriaceae* family) and bentonite (dioctahedral montmorillonite) are the first-ever products with an official anti-mycotoxin claim [112]. In particular, the strain BBSH 797 has been shown to modify the structure of trichothecenes (e.g., deoxynivalenol both in vitro and in vivo) through a biotransformation process that reduces their toxicity. Its use has been authorized in feed intended for all avian species and pigs [113]. In contrast, bentonite is used as an adsorbent material to reduce or diminish the toxicity of aflatoxin B1 in feeds for all animal species [114]. A recent review article reported that the use of bentonite clays is one of the most promising decontamination techniques that can be used by pisciculture to reduce the effects of aflatoxicosis [115]. In this regard, we suggest conducting more research in the field, including proof for other mycotoxins and species specificity, efficacy, and safety in fish.

## 6. Conclusions

This review emphasizes the growing concern over contamination by emerging mycotoxins, particularly those produced by *Fusarium* (ENNs, BEA) and *Alternaria* (AME, AOH), in the aquaculture supply chain. These toxins pose a potential risk to human health due to their presence in fish feeds and their ability to bioaccumulate in the tissues of commercially significant fish species.

Despite the growing body of research, significant gaps remain in our understanding of toxicity mechanisms, bioaccumulation, and the risks associated with chronic exposure to these compounds in fish/fish products and humans. Addressing these gaps is essential to refining risk assessments for both fish populations and consumers. In this context, we propose the following key research directions: (1) expanding in vitro and in vivo toxicity studies on emerging mycotoxins to elucidate their toxic mechanisms across different fish species and in humans, ultimately facilitating the establishment of maximum acceptable levels in feed and food; (2) developing new rapid, sensitive, and reproducible analytical strategies for detecting emerging mycotoxins in aquafeeds and fish tissues, with a focus on multi-mycotoxin methods suitable for study/understand the co-presence and synergism of different mycotoxins simultaneously; and (3) defining effective and sustainable mitigation strategies to control contamination in feeds and reduce exposure, with particular emphasis on biocontrol technologies based on microorganisms or natural compounds, such as volatile bioactive compounds, ITCs, probiotic bacteria, and yeast, to be brought to the attention of the competent authorities.

Moreover, future studies should also investigate the potential carry-over of emerging mycotoxins, also including insect-based aquafeed ingredients, as well as the impact of different aquaculture systems on fish exposure, particularly through water contamination by water-soluble mycotoxins in closed or semi-closed systems. These risks highlight the broader issue of emerging mycotoxins as a growing biosecurity concern in aquaculture. Addressing this challenge requires the implementation of a robust biosecurity plan that includes water quality management, the control of animal movements, disinfection protocols, feed safety measures, and staff training. Key preventive strategies, such as routine feed monitoring, the use of toxin binders, stringent raw material quality control, and proper feed storage, are essential to mitigate contamination risks. Understanding these dynamics is crucial to enhancing food safety and ensuring sustainable aquaculture practices.

## Figures and Tables

**Table 1:** Concentration and prevalence of emerging mycotoxins detected in fish feed samples and plant-based meals and oils intended for fish feed production.

Mycotoxin	Sample Type	Prevalence (%)	Mean Level (μg kg^−1^)	Range (μg kg^−1^)	Method	Reference
BEA	plant-based meals	12	416 ± 814	11–2400	^2^ LC-MS/MS ^1^ LOQ: 10 μg kg^−1^	[61]
	plant-based oils	10	16 ± 5	10–24		
	fish feed	4	16 ± 5	10–25		
ENNA	plant-based meals	1	<^1^ LOQ	90		
	plant-based oils	10	20 ± 12	10–38		
	fish feed	0.5	<^1^ LOQ	11		
ENNA1	plant-based meals	7	56 ± 49	15–140		
	plant-based oils	29	22 ± 7	11–37		
	fish feed	2	12 ± 27	10–16		
ENNB	plant-based meals	15	135 ± 186	11–530		
	plant-based oils	88	114 ± 119	12–450		
	fish feed	80	37 ± 35	10–250		
ENNB1	plant-based meals	10	78 ± 63	16–190		
	plant-based oils	65	38 ± 27	10–110		
	fish feed	27	18 ± 9	10–54		
BEA	fish feed	37	34.4	15.9–841.8	^3^ UHPLC-HRMS ^4^ LOD: 13–219 μg kg^−1^ ^1^ LOQ: 43–730 μg kg^−1^	[24]
ENNA	fish feed	3	<26.1	<26.1		
ENNA1	fish feed	5	<13.5	<13.5–23.8		
ENNB	fish feed	71	<38.8	<38.8–150.0		
ENNB1	fish feed	36	23.2	<12.9–43.5		
AOH	fish feed	30	<36.2	<36.2–43.3		
AME	fish feed	1	94.5	94.5		
BEA	fish feed	95	1.4	0.1–6.6	^2^ LC-MS/MS ^4^ LOD: 0.02–0.15 μg kg^−1^ ^1^ LOQ: 0.1–0.5 μg kg^−1^	[62]
ENNA	fish feed	100	0.9	0.6–3.4		
ENNA1	fish feed	100	1.1	0.3–6.5		
ENNB	fish feed	100	0.89	0.1–3.2		
ENNB1	fish feed	100	1.77	0.15–10		
BEA	fish feed	100	^5^ NM	0.5 ± 0.1–30 ± 2	^6^ HPLC-MS/MS ^4^ LOD: 0.05–0.2 μg kg^−1^ ^1^ LOQ: 0.16–0.7 μg kg^−1^	[25]
ENNA1	fish feed	44	^5^ NM	<^1^ LOQ–1.9 ± 0.5		
ENNB	fish feed	100	^5^ NM	0.6 ± 0.1–21 ± 1		
ENNB1	fish feed	100	^5^ NM	0.6 ± 0.2–7.9 ± 1.2		
BEA	fish feed	35.0	6.5	^5^ NM	^2^ LC-MS/MS ^4^ LOD: 0.03–0.3 μg kg^−1^ ^1^ LOQ: 0.1–0.01 μg kg^−1^	[63]
ENNA	fish feed	7.5	10.0	^5^ NM		
ENNA1	fish feed	19.0	2.7	^5^ NM		
ENNB	fish feed	46.9	6.6	^5^ NM		
ENNB1	fish feed	54.0	6.0	^5^ NM		
AOH	fish feed	40.7	5.0	^5^ NM		

^1^ LOQ: limit of quantification, ^2^ LC-MS/MS: liquid chromatography coupled to tandem mass spectrometry, ^3^ UHPLC-HRMS: Ultra-High-Performance Liquid Chromatography coupled with High-Resolution Mass Spectrometry, ^4^ LOD: limit of detection, ^5^ NM: not mentioned, ^6^ HPLC-MS/MS: High-Performance Liquid Chromatography coupled to tandem mass spectrometry.

**Table 2:** Concentration and prevalence of emerging mycotoxins detected in fish.

Mycotoxins	Range (μg kg^−1^)	Prevalence (%)	References
*Dicentrarchus labrax* (Sea Bass)			
ENNA	^1^ ND ^1^ ND		[62] [21]
ENNA1	1.7–6.9 1.7–6.9	50 50	[62] [21]
ENNB	1.3–44.6 1.3–12.8	90 90	[62] [21]
ENNB1	1.4–31.5 1.4–31.5	70 70	[62] [21]
BEA	^1^ ND ^1^ ND		[62] [21]
*Sparus aurata* (Sea Bream)			
ENNA	^1^ ND ^1^ ND ^1^ ND		[62] [21] [23]
ENNA1	2.1–7.5 2.1–7.5 ^1^ ND	30 30	[62] [21] [23]
ENNB	1.3–21.6 1.3–21.6 ^1^ ND	40 40	[62] [21] [23]
ENNB1	7.1–19 7.1–19 ^1^ ND	30 30	[62] [21] [23]
BEA	^1^ ND ^1^ ND ^1^ ND		[62] [21] [23]
*Salmo Salar* (Atlantic salmon)			
ENNA	^1^ ND ^1^ ND		[21] [23]
ENNA1	22–29 ^1^ ND	20	[21] [23]
ENNB	50–103 ^1^ ND	20	[21] [23]
ENNB1	56–94 ^1^ ND	20	[21] [23]
BEA	^1^ ND ^1^ ND		[21] [23]
*Oncorhynchus mykiss* (Rainbow trout)			
ENNA	^1^ ND		[21]
ENNA1	^1^ ND		[21]
ENNB	3.6	10	[21]
ENNB1	2.9	10	[21]
BEA	^1^ ND		[21]

^1^ ND: not detected.

**Table 3:** Overview of potential biocontrol strategies to mitigate fungal attacks and mycotoxin contamination in aquafeed.

Fungal Species	Mycotoxins	Biocontrol Strategy	Test Medium	Mitigating Effect	References
*P. verrucosum A. Flavus F. solani*	OTA AFs	Volatile organic compounds (BC344–2 strain)	Agar media	Significant inhibitory effect on fungal growth and alteration of their sporulating capacity.	[79]
*A. flavus*	AFs	Smoldering fumes (Cinnamon bark, Athl, Lilac leaves, plants)	Agar media Contaminated fish feed	All plants: strong antifungal activity observed; Cinnamon bark: complete inhibition of fungal spores in both media.	[104]
*A. parasiticus F. poae*	AFs BEA ENNs	Isothiocyanate (AITC)	Wheat flour	AITC inhibited all mycotoxins in a dose-dependent manner, showing a complete inhibition at 10 µL/L for 30 days.	[105]
-	BEA	Isothiocyanate (AITC)	PBS solution Wheat flour	PBS: time-dependent BEA reduction (20–100%); Wheat flour: dose-dependent BEA reduction (10–65%).	[106]
-	BEA	Isothiocyanate (BITC and PITC)	PBS solution Different kernels and flours	PBS: time-dependent BEA reduction (9–94%); Kernels and flours: dose-dependent BEA reduction (9–97%); PITC more effective than BITC.	[107]
-	BEA	Probiotic bacterial (13 different strains)	Growth medium	All bacterial strains showed a significant BEA reduction during fermentation (66–83%); *Lb. rhamnosus* showed the highest BEA reduction.	[109]
-	BEA	Yeast (nine different strains of *S. cerevisiae*)	Growth medium Corn flour	Growth medium: all strains showed BEA degradation in 48 h during fermentation (average of 86%); Corn flour: all strains showed BEA reduction (average of 71%); highest reduction was observed with the A42 strain (90.0 ± 3.1%).	[110]
-	BEA	Enzymes (four different strains of *S. cerevisiae*)	PBS solution Corn flour	PBS: time-dependent BEA reduction (83–99.3%); highest degradation with the A34 strain after 24 h; Corn flour: time-dependent BEA reduction (68–91%); highest degradation with the LO9 strain after 24 h.	[111]

## Data Availability

No new data were created or analyzed in this study.
